# Pregnant Women in Low- and Middle-Income Countries Require a Special Focus During the COVID-19 Pandemic

**DOI:** 10.3389/fgwh.2020.564560

**Published:** 2020-09-25

**Authors:** Chloe R. McDonald, Andrea M. Weckman, Julie K. Wright, Andrea L. Conroy, Kevin C. Kain

**Affiliations:** ^1^University Health Network-Toronto General Hospital, Toronto, ON, Canada; ^2^SAR Laboratories, Sandra Rotman Centre for Global Health, University Health Network-Toronto General Hospital, Toronto, ON, Canada; ^3^Toronto General Hospital Research Institute, University Health Network, Toronto, ON, Canada; ^4^Department of Laboratory Medicine and Pathobiology, Faculty of Medicine, University of Toronto, Toronto, ON, Canada; ^5^Tropical Disease Unit, Division of Infectious Diseases, Department of Medicine, University of Toronto, Toronto, ON, Canada; ^6^Department of Pediatrics, Indiana University School of Medicine, Indianapolis, IN, United States

**Keywords:** COVID-19, pregnancy, health access equity, low- and middle-income countries, antenatal care, adverse birth outcomes, sexual and reproductive health

## Introduction

As the novel coronavirus (SARS-CoV-2) pandemic continues to spread, some predict a disproportionate toll on low- and middle-income countries (LMICs), as it stresses already under-resourced health systems in densely populated regions (1). Two LMICs, Brazil and India, are among the top three countries by number of confirmed COVID-19 cases (2). While the reported prevalence of COVID-19 in sub-Saharan Africa is currently lower than reports from Asia, North America, and Europe, epidemiological modeling suggests that nearly a quarter of a billion people in sub-Saharan Africa may contract SARS-CoV-2 in the first year of the pandemic (3). The UN estimates up to 3 million COVID-19-related deaths in the region (4). Furthermore, the spread of SARS-CoV-2 in LMICs threatens to further increase the burden of adverse birth outcomes among the majority of global pregnancies.

**Figure 1:**
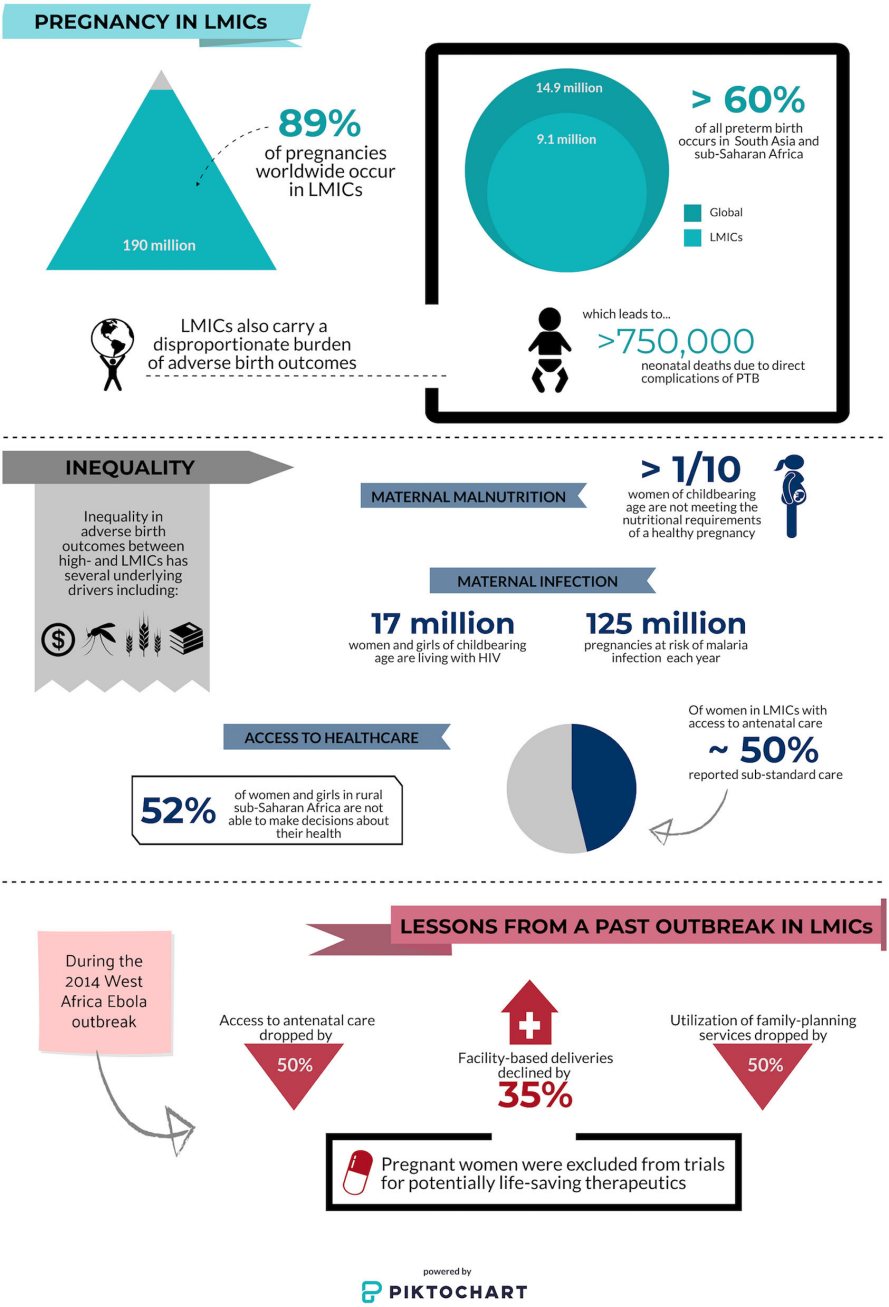
Infographic depicting inequalities in pregnancy outcomes and access to antenatal care at baseline and during a pandemic. Low- and middle-income countries (LMICs); preterm birth (PTB). Data sources (5–7, 9–13).

Globally, there are over 213 million pregnancies every year, of which an estimated 190 million (89%) occur in low resource settings where the risk of poor birth outcomes is highest (5). The contributing risk factors for these adverse outcomes are multifactorial: pregnant women in LMICs struggle to access antenatal care (6); an estimated 1 in 10 women in LMICs do not receive adequate nutrition in pregnancy (7); and the majority of pregnant women at risk of, or living with, malaria, HIV, and/or tuberculosis (TB) reside in LMICs (8, 9). High rates of these and other co-morbidities in pregnancy directly translate to adverse birth outcomes: more than 60% of children that are born preterm each year are born in sub-Saharan Africa and south Asia (e.g., India alone accounts for 23.6% of total global preterm births), accounting for over 750,000 deaths within the first month of life (Figure 1) (10).

COVID-19 is likely to influence maternal-child health in profound ways, from the physiological impact of the disease itself, to its indirect impacts on health systems, social, economic, and cultural structures, and by exacerbating pre-existing gender and healthcare access inequalities. Research is needed to identify the risks of COVID-19 in pregnancy, and its interplay with highly prevalent comorbidities already concentrated in LMICs including malnutrition, anemia, HIV, TB, and malaria. Identification and mitigation of both infectious and response-related barriers to health access and information for pregnant women during pandemics is essential for protecting the health of women and their children. Despite the current international focus on COVID-19, there remains an urgent need to direct research attention and resources to the impact of emerging infectious disease threats such as COVID-19 on pregnant women in LMICs.

## Evidence of the Impact of Respiratory Infection in Pregnancy

Very little information is currently available on the impact of COVID-19 in pregnancy. The first joint report published by the WHO and the Chinese Government mentions pregnancy twice in 40 pages (14). Much of the currently published literature contains conflicting information drawn from case reports and case-series with very small sample sizes. Although several original studies and systematic reviews suggest pregnant women are not at increased risk of severe clinical outcomes and that there is low risk of vertical transmission (15–19), one study reported seven maternal deaths out of nine cases in their multi-site COVID-19 case series (20). Increased rates of preterm birth and cesarean-section have also been reported (19, 21, 22). Due to the timing of the COVID-19 outbreak, most studies have reported on infection during the third trimester and there is little to no evidence of its impact in early pregnancy. Based on outcomes of pregnancies complicated by other severe respiratory infections, we hypothesize that more rigorous, pregnancy-focused research will reveal that pregnant women face an increased risk of poor clinical and birth outcomes during this COVID-19 pandemic.

Pregnant women have a higher risk of viral respiratory infection and are more likely to experience severe clinical symptoms (23). Both pandemic (e.g., H1N1) and seasonal influenza in pregnancy have been linked to severe maternal morbidity and increased risk of fetal death and preterm birth (24, 25). Pneumonia is also associated with increased risk of maternal morbidity, mortality, and poor birth outcomes. Co-existing maternal disease increases both the risk of infection as well as the risk of poor clinical outcomes (26). Therefore, as the COVID-19 pandemic continues, overlap with seasonal influenza and resulting co-infections will likely exacerbate morbidity and mortality in pregnancy. A rapidly growing body of evidence further indicates that infections during pregnancy, including respiratory infections such as influenza, are associated with increased risk of neurocognitive and neuropsychiatric disorders in exposed offspring (27). A review and a meta-analysis of coronavirus-spectrum infections reported increased preterm birth, miscarriage, preeclampsia, cesarean-section, and perinatal death in pregnant women with SARS, MERS, or COVID-19 (28, 29). However, the majority of data on coronavirus spectrum infections has been from Europe and North America. In LMICs, there is very little investigation into the impact of coronavirus infections on pregnancy despite the high prevalence of co-existing maternal conditions or co-infections, and barriers to quality antenatal care.

## Increased Risk for Women in Low- and Middle-Income Countries

During pregnancy, women's attendance at routine antenatal care visits results in high rates of exposure to health care environments. Consequently, women who continue to observe the recommended antenatal guidelines will be at increased risk for exposure to SARS-CoV-2. Furthermore, as health care systems become over-burdened by COVID-19, access and adherence to prenatal and obstetric care, as well as the quality of care, will suffer. Without timely intervention, pandemic-related restrictions on movement, reduced access to care, and economic constraints could lead to the reversal of important gains made in global antenatal care and maternal-child health. The 2014 Ebola outbreak in west-Africa provides a stark warning of the effect an infectious outbreak may have on already weak maternal-child health systems (11, 30). In Liberia, access to antenatal care plummeted by 50% and healthcare facility-based deliveries were reduced by 35% during the Ebola outbreak (11). Similar declines were also reported in Guinea, and by publication in 2017, had still not recovered to pre-outbreak levels (30). Preliminary evidence from both Uganda and Nepal indicates that even without a high COVID-19 burden, pandemic-related restrictions have already begun to impact maternal-child outcomes in LMICs, showing sharp declines in maternal facility deliveries (by 50% in Nepal), and increased maternal and neonatal mortality (31, 32). Although maternal-child outcomes with Ebola virus infection in pregnancy are more severe than existing evidence suggests for SARS-CoV-2 infection, these studies indicate immediate and lasting effects of emergent infectious diseases on vulnerable maternal-child healthcare systems in LMICs, and highlight the need for research and support to address this issue during the current COVID-19 pandemic.

The prevalence of medically complicated pregnancies is high among women living in LMICs. Women in LMICs carry a higher risk of infection with HIV, malaria, and/or tuberculosis compared to populations in high-income countries. Women of reproductive age in these regions are also more likely to have sickle cell disease, cardiac conditions (for example, rheumatic heart disease), and COPD due to indoor air pollution (33), conditions that increase the risk of developing severe COVID-19 (34). Furthermore, pregnant women in LMICs are at increased risk of having undiagnosed and/or sub-optimally managed gestational hypertension, pre-eclampsia, and gestational diabetes (35, 36). Hypertensive disorders and diabetes are both associated with an increased risk of severe COVID-19 in non-pregnant populations (34), but their impact on COVID-19 severity in pregnant women is not known. Furthermore, emerging data indicates the potential for long-lasting unintended consequences of governmental COVID-19 responses and COVID-19 related interruptions to maternal-child health interventions and critical public health programs (e.g., TB, HIV, and malaria diagnosis and treatment programs; nutritional interventions) on maternal morbidity and mortality in LMICs (32, 37, 38). The interplay of decreased health care access, increased prevalence of medical comorbidities, and the impact of SARS-CoV-2 exposure on pregnancy outcomes in LMICs warrants close surveillance and study in order to guide public health policies in the most at-risk regions.

Beyond antenatal care, pregnant and perinatal women will face psychosocial challenges related to stigma and/or social isolation, a lack of information or misinformation concerning neonatal care (e.g., appropriateness of breastfeeding with SARS-CoV-2 infection or suspected infection), and lack of or reluctance to access facility-based neonatal care services (e.g., for routine immunizations). Countries with strict restrictions on movement (e.g., banning public and private transport, curfews) have seen an impact on the ability of pregnant women to seek routine and/or emergency care, as well as increases in food insecurity and sexual and gender-based violence (32, 39). Challenges to providing antenatal and neonatal care during the COVID-19 outbreak will be further compounded if pregnant women and primary caregivers do not have access to up-to-date and accurate public health messaging to understand risks and recommendations.

Access to contraceptives is often limited in LMICs including sub-Saharan Africa and south Asia and the COVID-19 outbreak is likely to both disrupt global supply chains and prevent women from accessing providers of contraception. Many women will be isolated in domestic environments where they may not have input into family planning (Figure 1). Lessons from the Ebola crisis of 2014 indicate that widespread school closures will disproportionately affect girls of reproductive age, and lead to increased rates of sexual exploitation, sexual and gender-based violence, and forced marriage (39, 40). Compromising the sexual and reproductive health of women and girls means many are likely to experience pregnancy during the COVID-19 pandemic. Modeling estimates published by the Guttmacher Institute suggest that a 10% reduction in short and long-term contraceptive use could result in more than 15 million unintended pregnancies across 132 LMICs (41). As a result of gender inequality, the impact of a pandemic on sexual and reproductive health often goes unnoticed and unaddressed (12). Efforts to provide the means for pregnant women to safely access healthcare and healthcare providers are critical, as are efforts for widespread dissemination of public health policy and recommendations regarding other critical aspects of pregnancy (e.g., breastfeeding, immunization). Public and private health systems should craft responses to COVID-19 that address barriers to access and sexual and reproductive health outcomes in LMICs, in ways that protect the immediate health of pregnant women and inform future pandemic preparedness measures.

## Pregnant Women Deserve the Benefits of Drug and Vaccine Therapy

Pregnant women are almost uniformly excluded from clinical trials. Protecting vulnerable populations from risks associated with experimental therapies is essential and particularly important in LMICs where limited access to high-quality care creates additional vulnerabilities. However, the most at-risk populations also deserve to benefit from therapeutics that may improve outcomes. Many of the drugs being proposed for the treatment or chemoprophylaxis of COVID-19 have evidence-based safety profiles for use in pregnancy including lopinavir/ritonavir, remdesivir, and hydroxychloroquine (42–45). Yet, large government-funded clinical trials for treatment of COVID-19 (e.g., NIH-funded trials [NCT04280705, NCT04332991]) continue to list pregnancy as an exclusion criterion (46). The WHO Solidarity Trial [ISRCTN83971151] originally listed pregnancy in its exclusion criteria but has since removed it. As the WHO seems to have done, the ethics of excluding women from trials where they may benefit from treatments known to be safe in pregnancy needs to be carefully considered. During the 2013–2016 west-Africa Ebola outbreak, where mortality rates for pregnant women and their unborn children approached 100%, women were actively excluded from clinical trials of novel therapeutics (13). Pregnant women could not participate in clinical trials in the face of a life-threatening infection for which pregnancy increased their risk. This highlights the importance of understanding the unique impact of COVID-19 on pregnancy to assess the risk and benefits associated with novel treatments and vaccines.

As novel treatments and vaccines are developed and employed, we must consider why pregnant women continue to be excluded from trials by default, and when it is or is not appropriate to include them. Given the immediacy of the COVID-19 pandemic and the complexity of pharmacokinetics and pharmacodynamics in pregnancy, studies with therapies already known to be safe in pregnancy should be prioritized. To globally maximize benefits and health equity for pregnant and perinatal women, novel treatments should also be accessible to women living in the regions where most pregnancies occur (e.g., LMICs). These treatments should be effective, inexpensive, and easily accessible. Moreover, studies should also examine the impact of co-morbidities on therapeutic outcomes, including co-infection with HIV, malaria, and TB.

## Conclusions

As the global implications of the COVID-19 outbreak in LMICs begin to emerge, it is becoming increasingly clear that vulnerable populations will carry a disproportionate burden. Pregnant women in LMICs can face enormous obstacles to healthy birth outcomes for themselves and their unborn and newborn children and these barriers increase in the face of a global pandemic. As public health systems and the international medical research community focus resources on understanding COVID-19 and identifying therapeutics, the impact of infection in pregnancy and the unique health needs of pregnant women during a pandemic should not be neglected or passed over to be studied retrospectively. Pregnant women, including those in LMICs, deserve an immediate and enhanced focus during the COVID-19 outbreak to protect every woman and every child.

## Author Contributions

CM and KK: project conception, and oversight. CM, AW, JW, KK, and AC: research, analysis, writing, and editing. All authors: read and approved the final manuscript.

## Conflict of Interest

The authors declare that the research was conducted in the absence of any commercial or financial relationships that could be construed as a potential conflict of interest.
